# Two-Stage Design Method for Enhanced Inductive Energy Transmission with Q-Constrained Planar Square Loops

**DOI:** 10.1371/journal.pone.0148808

**Published:** 2016-02-18

**Authors:** Akaa Agbaeze Eteng, Sharul Kamal Abdul Rahim, Chee Yen Leow, Beng Wah Chew, Guy A. E. Vandenbosch

**Affiliations:** 1 Wireless Communication Centre, Universiti Teknologi Malaysia, Johor, Malaysia; 2 Intel Microelectronics, Halaman Kampung Jawa, Penang, Malaysia; 3 Department of Electrical Engineering, Katholieke Universiteit Leuven, Leuven, Belgium; Universidad Miguel Hernandez de Elche, SPAIN

## Abstract

Q-factor constraints are usually imposed on conductor loops employed as proximity range High Frequency Radio Frequency Identification (HF-RFID) reader antennas to ensure adequate data bandwidth. However, pairing such low Q-factor loops in inductive energy transmission links restricts the link transmission performance. The contribution of this paper is to assess the improvement that is reached with a two-stage design method, concerning the transmission performance of a planar square loop relative to an initial design, without compromise to a Q-factor constraint. The first stage of the synthesis flow is analytical in approach, and determines the number and spacing of turns by which coupling between similar paired square loops can be enhanced with low deviation from the Q-factor limit presented by an initial design. The second stage applies full-wave electromagnetic simulations to determine more appropriate turn spacing and widths to match the Q-factor constraint, and achieve improved coupling relative to the initial design. Evaluating the design method in a test scenario yielded a more than 5% increase in link transmission efficiency, as well as an improvement in the link fractional bandwidth by more than 3%, without violating the loop Q-factor limit. These transmission performance enhancements are indicative of a potential for modifying proximity HF-RFID reader antennas for efficient inductive energy transfer and data telemetry links.

## Introduction

The rapidly increasing proliferation of smart portable consumer devices has necessitated research into various ways to supplement battery charge. Wireless energy transfer through resonant inductive coupling mechanisms has been proposed as an effective alternative to conventional wired power delivery [1, 2]. Typically, an inductive energy transfer link consists of terminal loops, coils or spirals, whose magnetic fields are coupled, thereby facilitating the transfer of energy across the terminal structures. Various topologies have been studied in literature, ranging from two, three, and four loop links [3–5], and multi-terminal relay arrangements [6, 7]. There have also been numerous studies on enhancements to increase the efficiency of energy transfer between coupled terminals [8].

Increased device size is a valid concern when incorporating resonant inductive coupling mechanisms into portable devices. However, for devices already supporting proximity range High-Frequency Radio Frequency Identification (HF-RFID) capabilities at 13.56 MHz, the same infrastructure could be harnessed for inductive energy transfer within a few centimeters. This is possible due to the similarities in implementation between resonant inductive energy transfer and near-field non-radiative communications. However, HF-RFID systems are often not designed with the primary intent of efficient energy transmission [9]. The challenge, therefore, is to enhance the available infrastructure to support more efficient energy transmission.

The Q-factor is an important parameter of proximity range HF-RFID reader loop antennas, with an upper limit determined by the data rate requirements of the communication protocol employed [10]. Due to the inverse relationship between Q-factors and bandwidth, an important guideline in the design of HF-RFID links is for the reader loop antenna to have as low a Q-factor as possible within the prevailing design constraints [10]. Since the Q-factor increases with the number of turns, it is recommended, therefore, for the reader antenna to be implemented as a single-turn loop [10, 11]. However, this limitation in the number of turns would adversely affect the transmission performance of the reader antenna in an energy transfer application [5], and the extent of the reader interrogation zone in an HF-RFID interaction[12, 13]. Geometric techniques are discussed in [13, 14] to enable HF-RFID reader loops achieve longer interrogation distances without violating Q-factor limits. The methods are based on analytical closed-form expressions for loop performance parameters. However, these expressions are first-order approximations that ignore the complex parasitic interactions that occur when loops are inductively coupled[13]. For this reason, the physical realization of an analytically-derived transmission performance level cannot always be guaranteed, as seen in [15]. In the alternative, [16, 17, 18] propose a design-flow that harnesses the computational power of full-wave electromagnetic (EM) numerical simulations for multi-objective optimizations to achieve target Q-factors. However, the fully-automated design-flow requires additional automatic layout generation tools, without which manual modeling of all feasible designs in the search space is practically impossible.

Quite often, methods to improve performance by employing trade-offs implied in figures-of-merit are more practically relevant than a precise synthesis of globally optimal designs, and lead to quicker design times and lower costs. This is more especially so given the inevitable performance deviations arising from tolerances in component values used in the implementation of a design. A figure-of-merit to characterize the performance of two-terminal inductive coupling links is the product of the coupling coefficient *k* and the geometric mean of loop Q-factors[19, 20]. Higher numerical values of this figure-of-merit are indicative of good link transmission performance. This figure-of-merit also suggests that coupling coefficients and Q-factors equivalently determine the link transmission efficiency[21].

Consequently, the contribution of this paper is the assessment of a proposed hybrid two-stage geometric loop design method to improve link transmission performance. The method is based on coupling enhancement in a usage scenario in which Q-constrained planar square loops are employed in symmetric inductive energy transfer links. The first stage employs analytical models to achieve a performance level close to the desired objectives. In the second stage, the analytically realized loop layout is optimized using full-wave electromagnetic (EM) simulations to meet the desired objectives.

The rest of the paper is organized into 4 sections. Following this introductory section, an analytical modeling of planar square loops and link efficiency is presented. This is followed by a description of the proposed design method for enhancing inductive energy transmission without violating Q-factor constraints. Results obtained from the application of the design method to a test scenario are then discussed, after which the paper is concluded.

## Analytical Modeling

### Loop Model

**Fig 1:**
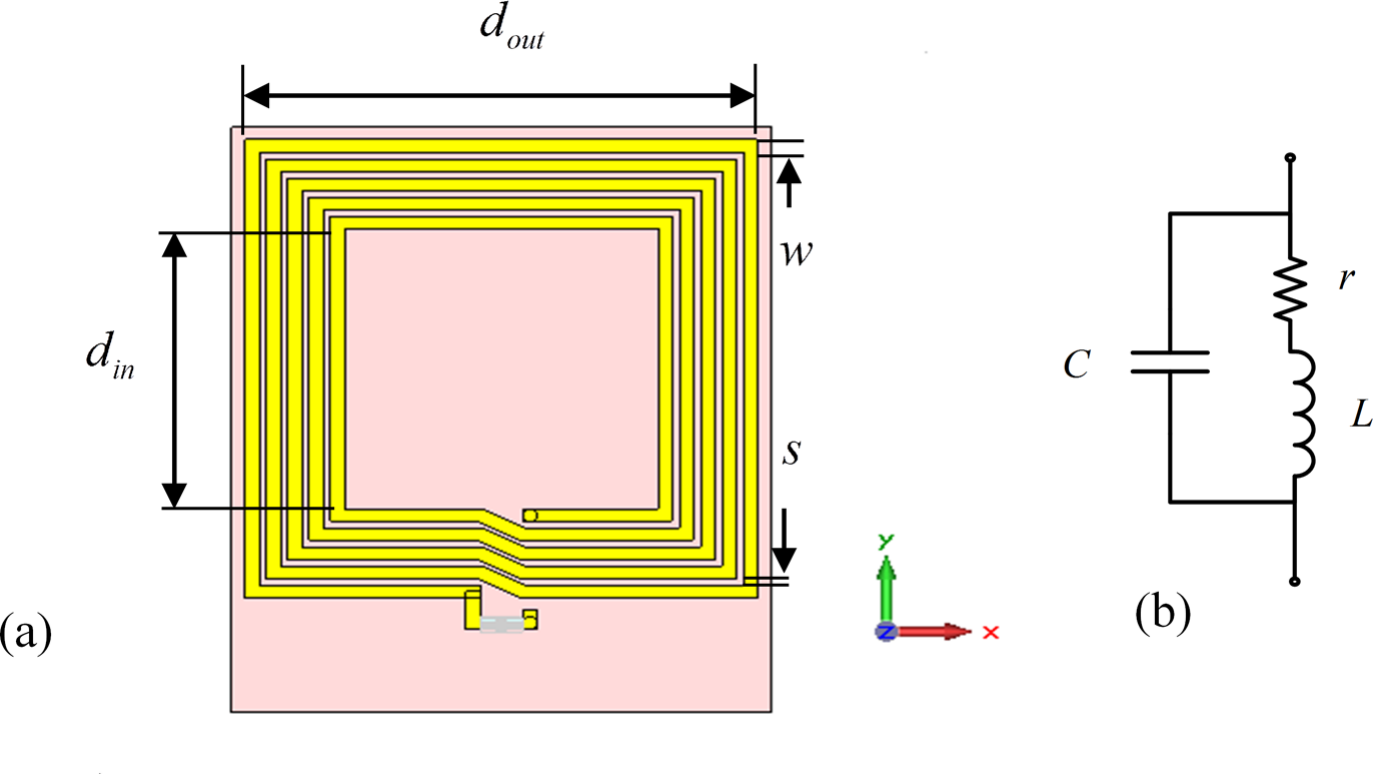
Planar square loop. (a) geometry (b) electrical model.

Fig 1 illustrates the general physical layout and equivalent circuit model of the planar square loops considered in this paper. The equivalent loop inductance *L*, resistance *R*, and parasitic capacitance *C* are functions of loop geometrical parameters.

Assuming an *N*-turn planar square loop is designed from a rectangular-sectioned square conductor trace on a dielectric substrate, as shown in Fig 1A, the loop self-inductance can be derived as

$$L=0.3175\mu N^{2}(d_{out}+d_{in})\left[\ln\left(2.07\left(\frac{d_{out}+d_{in}}{d_{out}-d_{in}}\right)\right)+0.18\left(\frac{d_{out}-d_{in}}{d_{out}+d_{in}}\right)+0.13{\left(\frac{d_{out}-d_{in}}{d_{out}+d_{in}}\right)}^{2}\right],$$ (1)

with *d*_*out*_ and *d*_*in*_ denoting the outer and inner side-lengths of the outermost and innermost loop turns respectively[22]. Also, *μ* is the conductor permeability. The outer side-length can be chosen such that the magnetic field excited by the loop at a required axial distance *z* is maximized. The magnetic field at *z* excited by a current *I* flowing through a single-turn square conductor trace can be calculated through the application of Biot-Savart’s law[23], leading to

$$H(z)=\frac{I}{2\pi \sqrt{z^{2}+0.5d_{out}^{2}}\left[{\left(z/d_{out}\right)}^{2}+0.25\right]}.$$ (2)

As shown in[5, 11], differentiating (Eq 2) with respect to *z*, and equating the result to zero provides an optimal relationship between *d*_*out*_ and *z* to maximize the axial magnetic field, which in this case is

$$d_{out}=z\sqrt{2+2\sqrt{5}}.$$ (3)

For the layout shown in Fig 1A, the inner side-length of an arbitrary *i*-th turn can be determined from the innermost turn side-length as

$$d_{i}=d_{in}+2\sum_{n=2}^{i}\left(w_{n-1}+s_{n-1}\right),$$ (4)

where *w*_*n*−1_ denotes the width of the (*n-1*)-th conductor trace, and *s*_*n*−1_ represents the spacing between the (*n-1*)-th and *n*-th conductor turns. Consequently, the outer side-length of the outermost turn can be derived as:

$$d_{out}=d_{in}+2\left(w_{1}+\sum_{i=2}^{N}\left(w_{i}+s_{i-1}\right)\right),$$ (5)

where *w*_*i* = *N*_ is the width of the outermost conductor trace.

The parasitic capacitance is approximated as a parallel combination of the capacitances forming between loop conductor turns as a result of air gaps between the turns, and the substrate dielectric [5]. Consequently, assuming the use of an FR4 substrate board,

$$C\approx \frac{tl_{g}}{\Delta }\left(0.9\varepsilon_{air}+0.1\varepsilon_{subs}\right),$$ (6)

where *ε*_*air*_ and *ε*_*subs*_ are the absolute permittivities of air and the FR4 substrate board, respectively [5]. Δ refers to the average spacing between turns, which is calculated as

$$\Delta =\frac{1}{N-1}\sum_{i=1}^{N-1}s_{i}.$$ (7)

$l_{g}$ is the total length of the air gap between loop turns, and is calculated using

$$l_{g}=\frac{1}{2}\sum_{i=1}^{N-1}d_{i}+d_{i+1}.$$ (8)

Taking into account the skin effect at higher frequencies, the loop resistance can be approximated as [5]:

$$R=\frac{4\rho \sum_{n=1}^{N}d_{n}/w_{n}}{\delta \left(1-e^{-t/\delta }\right)},$$ (9)

where

$$\delta =\sqrt{\frac{\rho }{\pi \mu f}}.$$ (10)

*t* and *ρ* refer to the vertical thickness, and the resistivity of the loop conductor turns, respectively, at the frequency *f*. However, the actual resistance is expected to be slightly higher than predicted by (Eq 6), due to the proximity effect[24].

On the basis of the electrical model for the planar square loops [5], shown in Fig 1B, the loop Q-factor can be calculated as:

$$Q=\frac{\omega }{R}\left(L-R^{2}C-\omega^{2}L^{2}C\right),$$ (11)

where *ω* = 2*πf*, and with (Eq 1) and (Eq 10) enabling the computation of the equivalent circuit values at the frequency *f*.

### Link Efficiency

As shown in [5], a mutual inductance develops between a pair of similar and axially aligned planar square loops, separated by a distance *z*, which can be written as:

$$L_{m}=1.1\sum_{i=1}^{N_{tx}}\sum_{j=1}^{N_{rx}}\left(\mu \sqrt{\frac{d_{tx(i)}d_{rx(j)}}{2}}\left[\left(\frac{2}{\kappa }-\kappa \right)K\left(\kappa \right)-\frac{2}{\kappa }E\left(\kappa \right)\right]\right),$$ (12)

with

$$\kappa =2{\left(\frac{d_{tx(i)}d_{rx(j)}}{{\left(d_{tx(i)}+d_{rx(j)}\right)}^{2}+z^{2}}\right)}^{\frac{1}{2}}.$$ (13)

*K*(*κ*) and *E*(*κ*) are the complete first and second kind elliptic integrals, and the lengths *d*_*tx*(*i*)_ and *d*_*rx*(*j*)_ are derived using (Eq 4). The subscripts '*tx*' and '*rx*' denote the transmit and receive planar loops respectively.

In order to quantify the magnetic coupling between paired planar loops, this mutual inductance can be normalized to the shared inductance of the individual loops, yielding the coupling coefficient defined as:

$$k=\frac{L_{m}}{L}.$$ (14)

**Fig 2:**
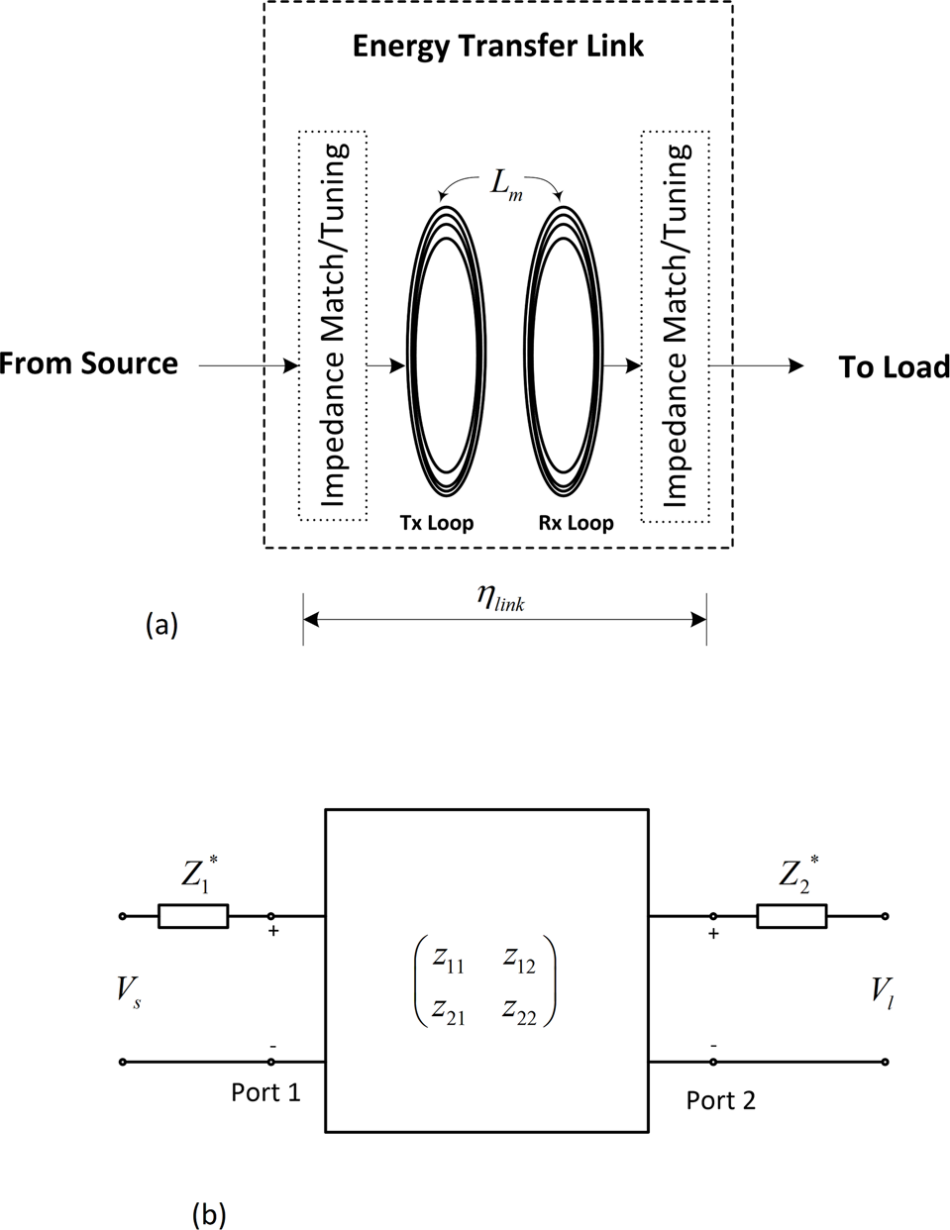
Inductive energy transfer link model. (a) schematic (b) two-port network model.

Fig 2 is a representation of an inductive energy transfer link. $Z_{1,2}^{*}$ represent equivalent impedances required to ensure zero power reflection at ports 1 and 2. While Re(Z1,2*) realize the maximum power transfer criterion, Im(Z1,2*) tune the link to the required frequency of operation *f*.

The coefficients of the impedance matrix in Fig 2B can be defined as:

$$z_{11}=\frac{R_{1}+j\omega L_{1}}{1-\omega^{2}L_{1}C_{1}+j\omega R_{1}C_{1}}$$ (15)

$$z_{12}=j\omega L_{m},$$ (16)

$$z_{21}=j\omega L_{m},$$ (17)

$$z_{22}=\frac{R_{2}+j\omega L_{2}}{1-\omega^{2}L_{2}C_{2}+j\omega R_{2}C_{2}}.$$ (18)

The subscripts in (Eqs 15–18) denote the corresponding ports. Equations (Eq 16) and (Eq 17) are based on an assumption of a negligible capacitive coupling between the pair of coupled loops. Simultaneous conjugate matching of the inductive energy transfer link is achieved by meeting the criterion [20]

$$Z_{1}^{*}=z_{11}-\frac{z_{12}z_{21}}{z_{22}+Z_{2}},$$ (19)

$$Z_{2}^{*}=z_{22}-\frac{z_{12}z_{21}}{z_{11}+Z_{1}}.$$ (20)

If the link is tuned to the required resonance frequency *f*, then Im{*Z*_1_} = Im{*Z*_2_} = 0, while $Z_{1}=Z_{1}^{*}$, and $Z_{2}=Z_{2}^{*}$. Consequently, the transmission matrix of the inductive energy transfer link at resonance can be defined in terms of its impedance matrix as

(ABCD)=(1+Re{Z1}z12Re{Z1}+Re{Z2}+Re{Z1}Re{Z2}z121z121+z12Re{Z2}). (21)

Similarly, the scattering parameter matrix can be defined in terms of this transmission matrix, so that the transmission coefficient of the scattering parameter matrix, *s*_21_, can be defined as [25]

s21=2Re{Z1}Re{Z2}ARe{Z2}+B+CRe{Z1}Re{Z2}+DRe{Z1}. (22)

Substituting (Eqs 15–21) in (Eq 22), and solving for |*s*_21_|^2^, leads to

$${\left|s_{21}\right|}^{2}=\frac{\omega^{2}L_{m}{}^{2}\left(L-R_{1}{}^{2}C_{1}-\omega^{2}L_{1}{}^{2}C_{1}\right)\left(L-R_{2}{}^{2}C_{2}-\omega^{2}L_{2}{}^{2}C_{2}\right)}{R_{1}R_{2}{\left(1+\sqrt{1+\frac{\omega^{2}L_{m}{}^{2}\left(L-R_{1}{}^{2}C_{1}-\omega^{2}L_{1}{}^{2}C_{1}\right)\left(L-R_{2}{}^{2}C_{2}-\omega^{2}L_{2}{}^{2}C_{2}\right)}{R_{1}R_{2}}}\right)}^{2}}.$$ (23)

However, from (Eq 11) and (Eq 14), the following relationship can be deduced:

$$\frac{\omega^{2}L_{m}{}^{2}\left(L-R_{1}{}^{2}C_{1}-\omega^{2}L_{1}{}^{2}C_{1}\right)\left(L-R_{2}{}^{2}C_{2}-\omega^{2}L_{2}{}^{2}C_{2}\right)}{R_{1}R_{2}}=k^{2}Q_{1}Q_{2}.$$ (24)

Hence, (Eq 23) becomes

$${\left|s_{21}\right|}^{2}=\frac{k^{2}Q_{1}Q_{2}}{{\left(1+\sqrt{1+k^{2}Q_{1}Q_{2}}\right)}^{2}},$$ (25)

which, when expressed as a percentage, is the link transmission efficiency *η*_*link*_ [20, 26], illustrated in Fig 2A.

## Design Method

**Fig 3:**
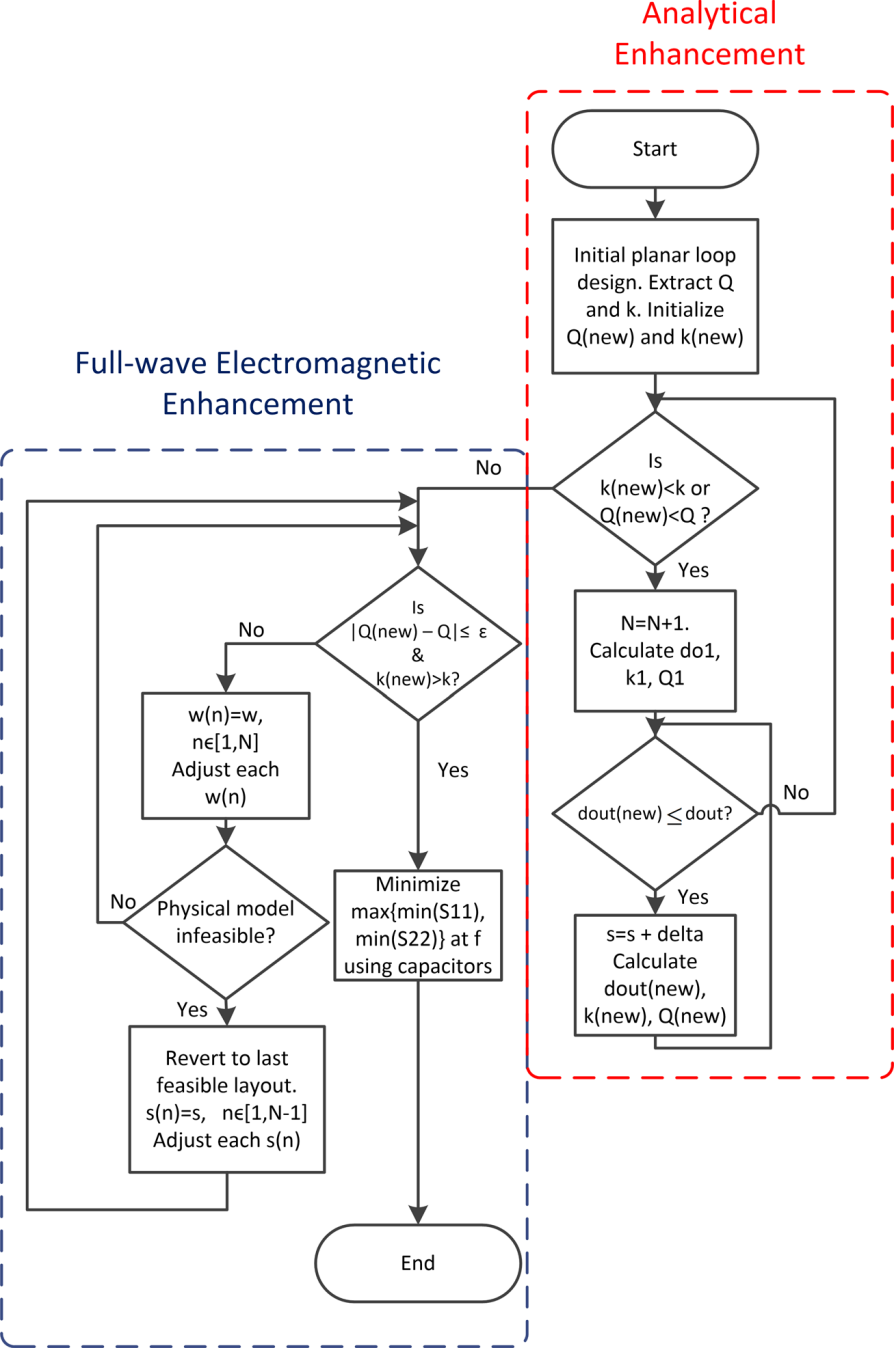
Proposed two-stage design method.

The analytical modeling of the previous section basically reveals that a geometric enhancement of transmission performance of a link depends on five geometric variables, namely *d*_*in*_, *d*_*out*_, *N*, *w*_*n*_, and *s*_*n*−1_, *n* ∈ [1,*N*]. For this study, *d*_*out*_ is determined from the required operating distance, as shown in (Eq 3). *d*_*in*_, on the other hand, depends on assumed installation requirements. Consequently, the design method is based on the manipulation of three variables, namely *N*, *w*_*n*_, and *s*_*n*−1_, *n* ∈ [1,*N*], in order to enhance link transmission performance through increased coupling only, if loop Q-factors are restricted. To this end, the proposed two-step synthesis flow is shown in Fig 3. The first step applies (Eqs 1–14) to analytically determine the number of turns that places the performance as close to the desired objectives of enhanced coupling and matched Q-factor as possible. The resulting link transmission efficiency is calculated by substituting the realized Q-factors and coupling coefficient in (Eq 25). In the second stage, a model of the analytical realized loop configuration is simulated in a full-wave EM solver, and optimized for the best values of *w*_*n*_, and *s*_*n*−1_ that match the Q-factor limit while still maintaining an enhanced coupling coefficient. The criterion for testing the Q-factor match is |*Q*(*new*)−*Q*| ≤ *ε*, where *Q* is the Q-factor limit, *Q*(*new*) is the Q-factor of the modified loop, and *ε* is a suitable tolerance level.

**Fig 4:**
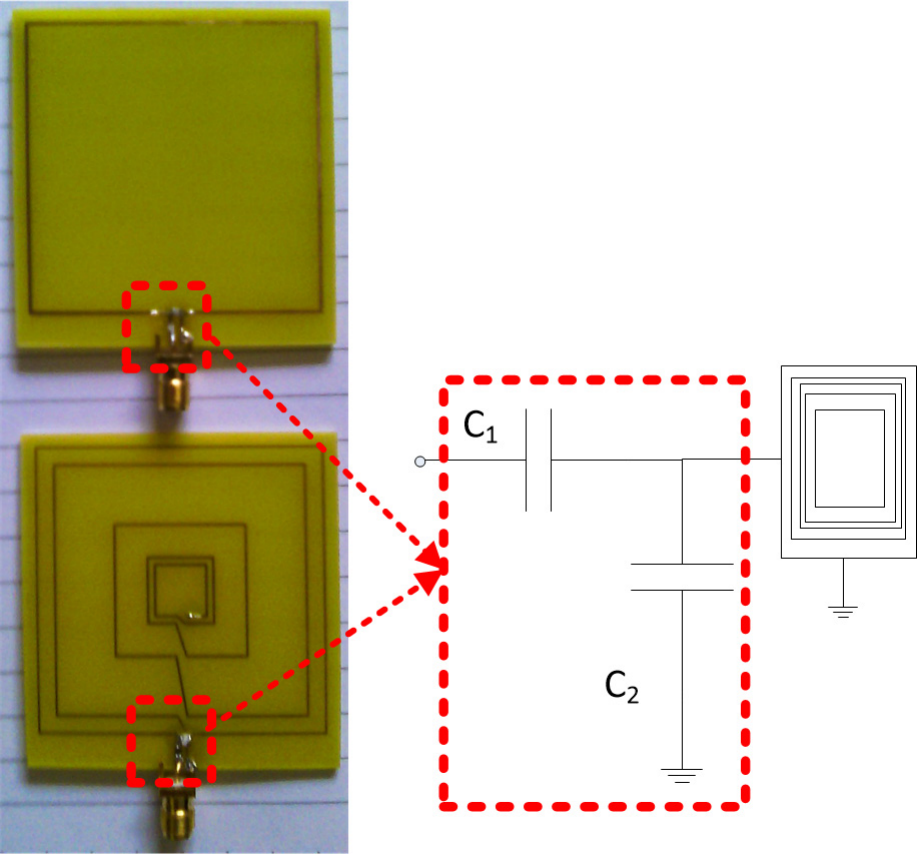
Fabricated initial (top) and modified (bottom) planar square loops.

Once the criteria of enhanced coupling and Q-factor matching have been met by the modified loop design, impedance matching networks are designed using scattering parameters at both terminals to bring the link into resonance[27]. To this end, the coupled link is modeled as a two-port network, and capacitive L-match networks, shown in Fig 4, are designed in the EM software tool to achieve impedance matching and resonance conditions. The impedance matching algorithm is given as:

$$\begin{array}{l} \text{while}\;g(C_{1},C_{2})>0\\ \;g(C_{1},C_{2})=\max\left\{{\left|s_{11}\left(C_{1},C_{2}\right)\right|}_{f},{\left|s_{22}\left(C_{1},C_{2}\right)\right|}_{f}\right\}\\ \;\min_{C_{1},C_{2}\in ℜ}g(C_{1},C_{2})\\ \text{end} \end{array}.$$ (26)

*g* is a cost function, which, at each iteration, takes its value from the larger of the magnitudes of the input and output port voltage reflection coefficients, *s*_11_ and *s*_22_, respectively, at the frequency *f*. *s*_11_, *s*_22_ and *g* are functions of the capacitors C_1_ and C_2_ employed in the L-matching circuits at both terminals. Any suitable inbuilt search algorithm in the EM solver can be used to minimize the cost function *g* by adjusting the capacitance values C_1_ and C_2_ until *g* = 0. The link transmission efficiency can then be derived from the *s*_21_ parameter value corresponding to this point of convergence.

## Results and Discussion

**Fig 5:**
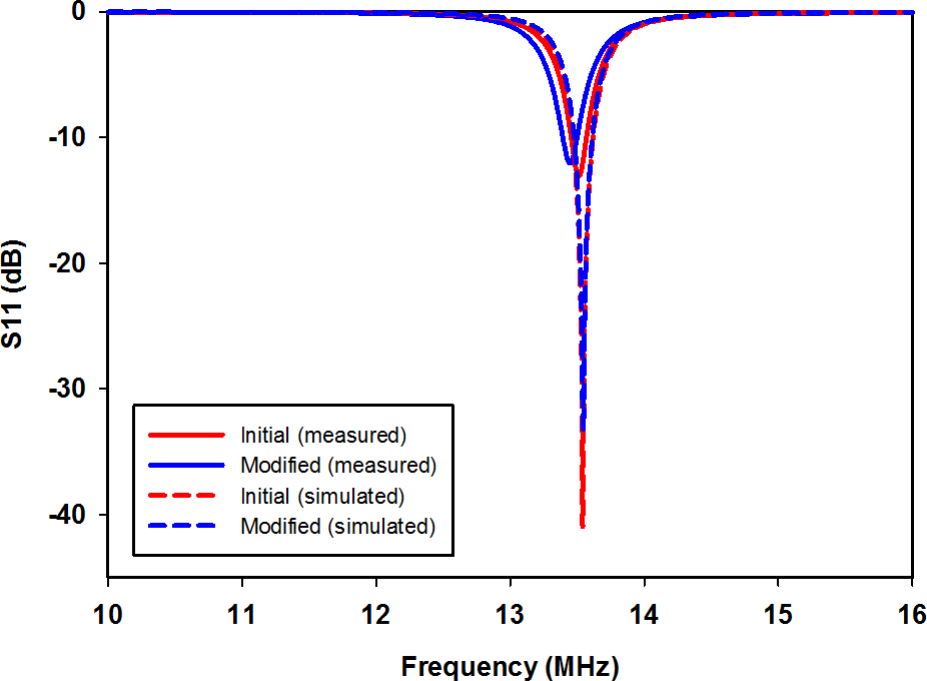
Reflection coefficients of planar square loops.

**Fig 6:**
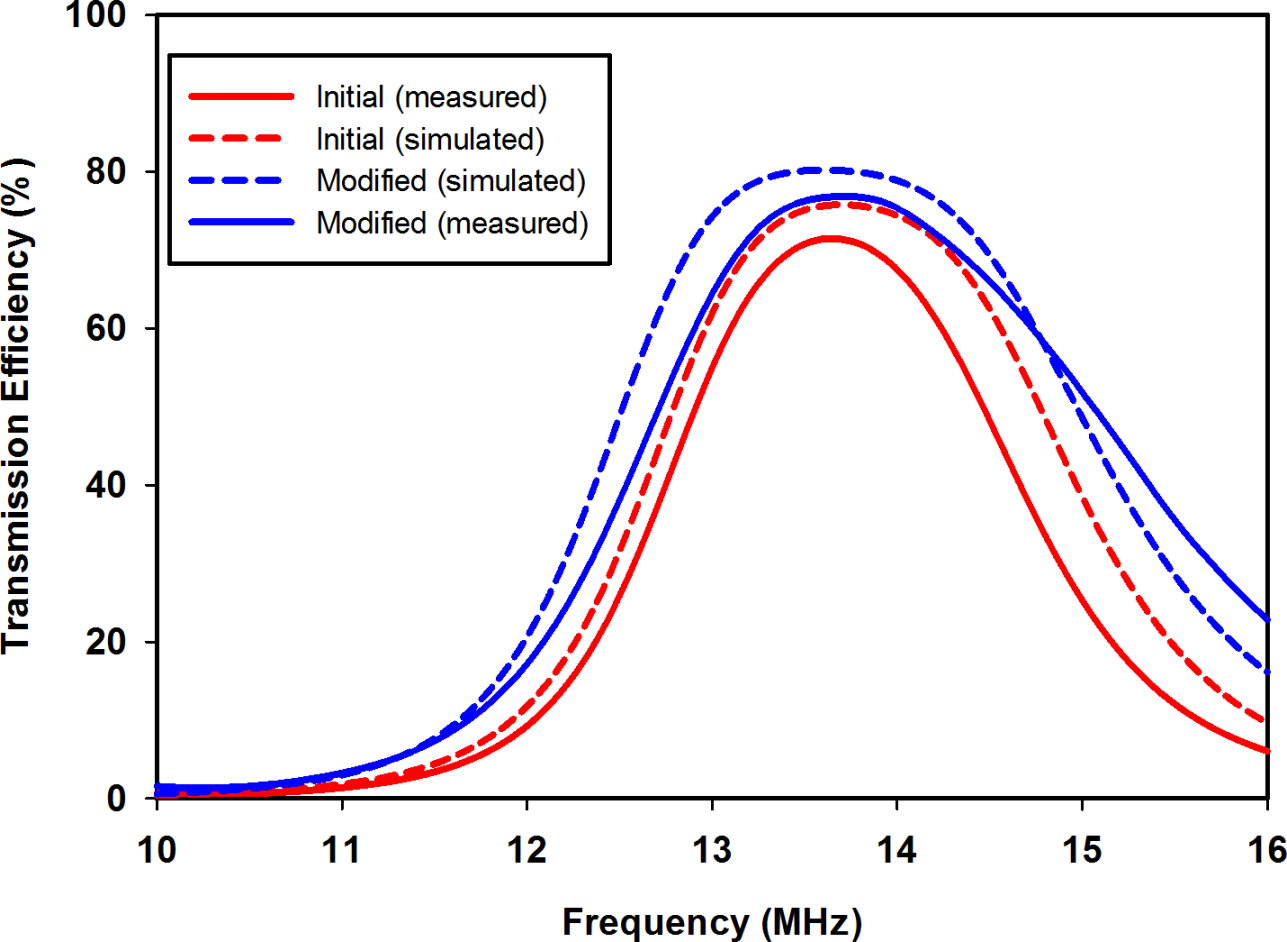
Link transmission efficiencies between planar square loops.

**Fig 7:**
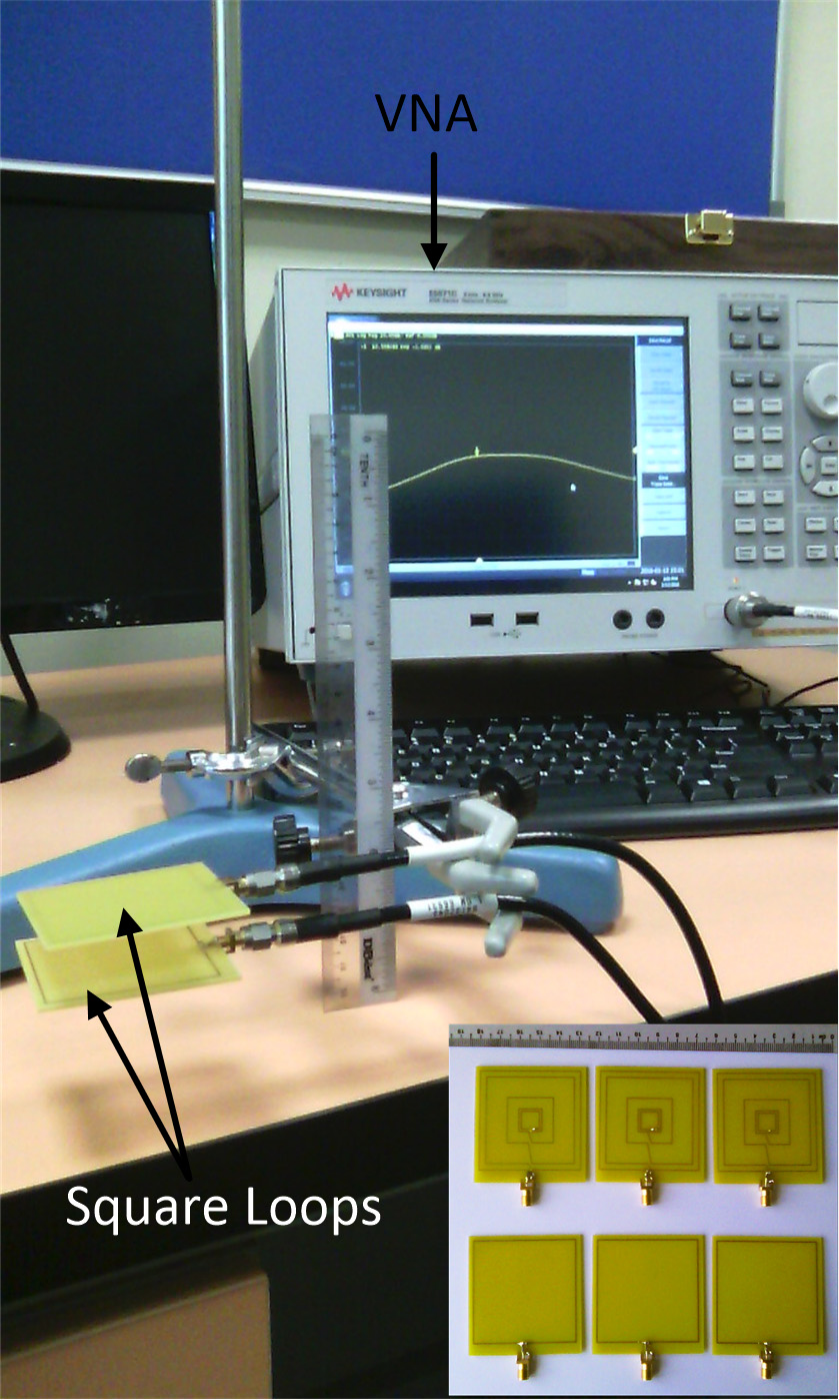
Experimental test stand.

The proposed synthesis flow was assessed by application in a test scenario that modifies an initial single-turn planar square loop for a 13.56 MHz symmetric link operating at a distance *z* = 20 mm. The initial and modified planar square loops were designed for implementation on low-cost FR4 substrate boards, with a relative permittivity of 4.7. The first-stage analytical enhancement was performed using MATLAB, while CST Microwave Design Studio was employed for the second-stage full-wave EM enhancement. The full-wave EM simulations were based on the finite integration technique, while the EM model optimization, and impedance matching network design were realized using the inbuilt Trust Region Framework algorithm[28]. Table 1 contains the realized geometric and performance parameters for the initial and modified loops in the test scenario. The computed capacitance values for the impedance matching networks are listed in Table 2. Fig 4 shows the physical structure of the realized planar square loops. To compare the standalone bandwidth potentials of the loop designs, single planar square loops of the initial and modified design were separately matched using capacitive L-match arrangements, and their reflection coefficients obtained, as shown in Fig 5. The link transmission efficiencies of the initial and modified loops, shown in Fig 6, allow for a comparison of the transmission efficiency at the operating frequency, and bandwidth potential of the realized inductive energy transfer links. Experimental values of the loop standalone reflection coefficients and link transmission efficiencies were obtained through s-parameter measurements using a vector network analyser (VNA). The experimental test stand is shown in Fig 7.

**Table 1:** Loop Geometric and Performance Parameters.

Parameter	Initial Loop		Modified Loop	
	Analytical Model	Full-wave Simulation	Analytical Enhancement	Full-wave EM Enhancement
*N*	1	1	5	5
*k*	0.0890	0.1160	0.1162	0.1451
*Q*	40.1978	62.60	54.4043	62.61
*d*_*out*_	50.9 mm	50.9 mm	50.9 mm	50.6 mm
*d*_*in*_	49.9 mm	49.9 mm	8 mm	8.7 mm
*t*	0.035 mm	0.035 mm	0.035 mm	0.035 mm
*s*_*n-1*_	-	-	4.7 mm	[0.95, 5.45, 10.2, 2.85] mm
*w*_*n*_	0.5 mm	0.5 mm	0.5 mm	[0.30, 0.30, 0.30, 0.30, 0.30] mm
*η*_*link*_ (%)	57.58%	75.0%	72.97%	80.1%
*Measured η*_*link*_ (%)	71.16%		76.68%	

**Table 2:** Impedance Matching Capacitance Values.

**Standalone Match**	**Initial Loop**	**Modified Loop**
**C**_**1**_**(pF)**	56.99 (56^a^)	23.34 (24^a^)
**C**_**2**_**(pF)**	724.84 (723^a^)	140.56 (140^a^)
**Symmetric Link Match**	**Initial Loop**	**Modified Loop**
**C**_**1**_**(pF)**	176.72 (177^a^)	76.13 (76^a^)
**C**_**2**_**(pF)**	592.13 (593^a^)	90.57 (91^a^)

^a^ Available off-the-shelf values

With respect to Table 1, the value of *d*_*out*_ is the outer side-length dimension of the initial loop that maximizes the excited magnetic field at a chosen operating range of 20 mm according to (Eq 3). This value dimension represents an outer size limit, to ensure that the modified design is not larger than the initial design. On the other hand, the inner length dimension *d*_*in*_ is constrained to a minimum of 8 mm, which assumes the need for the installation of a chip at the loop center [5]. In the analytical enhancement stage, the loop modification is achieved with *d*_*in*_ at this minimum. However, the full-wave EM enhancement slightly increases the value of *d*_*in*_ to 8.7 mm. Also, the analytical enhancement step employs uniform turn spacing and widths, while the full-wave EM enhancement results in varying turn spacings and, in this case, uniform turn widths. These dimensions are represented in arrays, starting from the innermost turn spacing and width to the outermost. The initial and modified designs are implemented using the same FR4 substrate material, so that the thickness of the conductor strip on the dielectric substrate *t* is same for both designs.

It also can be observed from Table 1 that, for the initial loop, the analytically computed Q-factor, coupling coefficient, and transmission efficiency values are less than their corresponding full-wave EM simulated values. This is due to the fact that the approximate electrical model and analytical expressions used for modelling the loops do not account for the complex parasitic and radiation effects inherent in an inductively coupled link. Consequently, the real-life performance of designs synthesized solely from analytical models cannot be guaranteed. However, the Q-factor and coupling coefficient results show that the analytical stage has enabled a loop modification with a performance close to the desired enhanced coupling within Q-factor limits.

From Fig 5, it can be observed that the simulated reflection coefficients at 13.56 MHz are -30.71 dB and -20.21 dB for the standalone initial and modified loop designs, respectively. The measured standalone reflection coefficients, however, are -12.99 dB at 13.52 MHz for the initial design, and -12.03 dB at 13.45 MHz for the modified design. The higher measured reflection coefficient values and slight shifts in resonance frequency are due to fabrication imperfections, and the tolerances of the off-the-shelf capacitors used to implement the L-matching circuits, which are listed in Table 2. Nonetheless, the equivalence in the Q-factor values of the initial and modified loop results in similar standalone bandwidth characteristics for the initial and modified loops. The simulated -10 dB bandwidths for the initial and modified loops are both 156 kHz, while the measured value is 120 kHz for both loops. The fact that the modified loop has the same bandwidth as the initial loop confirms that the loop modification has been achieved without an alteration of the standalone bandwidth performance. The effect of an enhanced coupling resulting from the use of the modified loop is seen in Fig 6. The simulated peak transmission efficiency at 13.56 MHz has been increased by 5.1%, from 75.0% to 80.1%. Similar trends are observed from measurement results, with the transmission efficiency increased from 71.16% to 76.68% at 13.56 MHz. The increased coupling from the modified loop has also led to a larger link transmission bandwidth as compared to the initial design. The simulated -3 dB link fractional bandwidth, in this case, is increased from 17.52% to 20.77%, while an increment from 15.14% to 20.89% was observed in the measured values. Differences between simulations and measurements for the peak transmission efficiency values again are due to fabrication inaccuracies, and tolerances of the off-the-shelf capacitors used to realize the L-matching circuits.

## Conclusion

This paper has used a two-stage design method to design Q-constrained square loops to achieve enhanced inductive energy transmission performance. The two-stage design method effectively reduces the search space required for obtaining a geometric layout for an enhanced design. The first stage, which is analytical, is used to obtain the relatively more straightforward number of turns required to enhance the coupling performance, with a uniform spacing employed to move the Q-factor level close to the required limits. The second stage employs optimizers in a full-wave EM simulation environment to fine-tune the coupling enhancement, while meeting the prescribed Q-factor limits. In practice, this means that the enhancement of transmission performance is reached without a change in the Q-factor. The two-stage design method, when applied to a test-case, led to a more than 5% increase in the transmission efficiency, and a more than 3% increase in the fractional bandwidth of the inductive coupling link, without a change in the standalone bandwidth potential of the synthesized loops.
